# The impact of an employee wellness programme in clothing/textile manufacturing companies: a randomised controlled trial

**DOI:** 10.1186/1471-2458-13-25

**Published:** 2013-01-11

**Authors:** Naila Edries, Jennifer Jelsma, Soraya Maart

**Affiliations:** 1 Division of Physiotherapy, Department of Health and Rehabilitation Sciences, University of Cape Town, Anzio Road, Cape, South Africa

**Keywords:** Musculo-skeletal disorders, Employee wellness, Cognitive behaviour therapy, Occupational health

## Abstract

**Background:**

The prevalence of health risk behaviours is growing amongst South African employees. Health risk behaviours have been identified as a major contributor to reduced health related quality of life (HRQoL) and the increased prevalence of non-communicable diseases. Worksite wellness programmes promise to promote behaviour changes amongst employees and to improve their HRQoL. The aim of this study was to evaluate the short-term effects of an employee wellness programme on HRQoL, health behaviour change, body mass index (BMI) and absenteeism amongst clothing and textile manufacturing employees.

**Methods:**

The study used a randomised control trial design. The sample consisted of 80 subjects from three clothing manufacturing companies in Cape Town, South Africa. The experimental group was subjected to a wellness programme based on the principles of cognitive behaviour therapy (CBT) as well as weekly supervised exercise classes over six weeks. The control group received a once-off health promotion talk and various educational pamphlets, with no further intervention. Measurements were recorded at baseline and at six weeks post-intervention. Outcome measures included the EQ-5D, Stanford Exercise Behaviours Scale, body mass index and absenteeism.

Data was analysed with the Statistica-8 software program. Non-parametric tests were used to evaluate the differences in the medians between the two groups and to determine the level of significance. The Sign test was used to determine the within group changes. The Mann–Whitney U test was used to determine the difference between the two groups.

**Results:**

At six weeks post intervention the experimental group (39 subjects) demonstrated improvement in almost every parameter. In contrast, apart from an overall decrease in time off work and a reduction in BMI for all study participants, there was no significant change noted in the behaviour of the control group (41 subjects). Seventy percent of the experimental group had improved HRQoL EQ-5D VAS scores post intervention, indicating improved perceived HRQoL. In comparison, only 58% of the control group had improved HRQoL EQ-5D VAS scores post intervention. There was no significant difference between the two groups at baseline or at six weeks post intervention.

**Conclusion:**

An employee wellness programme based on the principles of CBT combined with weekly aerobic exercise class was beneficial in improving the perceived HRQoL and changing health-related behaviours of clothing manufacturing employees. However, it cannot be concluded that the EWP was more effective than the once off health promotion talk as no significant changes were noted between the two groups at 6-weeks post intervention.This trial has been registered with ClinicalTrials.gov (trial registration number NCT01625039).

## Background

Reduced worker productivity and increased absenteeism related to musculoskeletal pain complaints are commonly encountered within the work force. Musculoskeletal disorders can result in lengthy periods off work and increased utilisation of health-care services [1,2]. Musculoskeletal injuries such as lower back pain (LBP) and repetitive strain injuries (RSI) have been reported as having a high prevalence amongst clothing and textile manufacturing workers [3-5]. This may be attributed to occupational exposure to repetitive work tasks, prolonged sedentary postures, heavy lifting, intensive-physical labour and long working hours. Several studies have also identified behavioural factors such as obesity, physical inactivity, poor diet and substance abuse as being closely associated with the development of LBP and occupation related RSI [6,7].

In South Africa, the 1998 South African Demographic and Health Survey reported that as many as 30% of all reported occupational injuries in South Africa were musculoskeletal in nature. In addition, the prevalence of health risk factors amongst the adult population is high. The 2002 cycle of the same survey reported nearly 50% of men and women in the general population to be physically inactive, and 55% of the females to be overweight [8].

Recent studies have reported on the positive effects of supervised exercise programmes for managing various health conditions [9-11]. The growing research in behavioural medicine suggests that cognitive behaviour therapy (CBT) has a significant role to play in the management of chronic pain conditions[12]. The theory underpinning CBT is that both patients’ perception of their condition and their understanding of pain directly influences their self-efficacy levels [13]. CBT based programmes aim to equip patients with the knowledge, behavioural ability and cognitive skills needed in order to improve their state of health and functional abilities[12].Worksites have been identified as the ideal setting for health promotion programmes as workers spend the majority of their day at work. Several international studies have reported improved self-efficacy and fitness levels following participation in a worksite exercise intervention programme [14,15]. Limited literature is available as to the effects of worksite wellness programmes implemented in Africa. A study performed in South Africa within an electricity supply company has reported on the positive effect of worksite health promotion and exercise programme [16]. Many companies in developed nations have started implementing various worksite health promotion progammes.The benefits of these worksite health promotion initiatives have been reported in many studies [14,15,17-20]. These workplace health promotion programmes ranged from web-based councelling, print materials, group activities, to supervised exercise classes. A review by Harden et al. [21] suggests that the effectiveness of any workplace health promotion intervention depends on committment and support from employers, as well as motivation of employees to participate.

In the light of the health and economic burden of musculo-skeletal disorders in the clothing industry and the reported positive effects of supervised exercise programmes and CBT, an employee wellness programme was developed incorporating these elements and tested in three clothing manufacturing companies in the Cape Town Metropolitan region. The aim of this study was therefore to evaluate the short-term effects of an employee wellness programme on HRQoL, health behaviour change, body mass index (BMI) and absenteeism amongst clothing and textile manufacturing employees.

The objectives of the study were:

· To compare the demographics of the two groups in terms of gender, age, level of education and skill using frequency distributions to ensure equivalence.

· To compare the health status of the sample groups with regard to HRQoL, exercise behaviours and BMI to ensure equivalence.

· To determine if an employee wellness programme has an impact on health-related quality of life, using the EQ-5D.

· To assess the short-term effects of a wellness programme on physical activity, using the Stanford Exercise Behavior Scale.

· To determine whether the implementation of a wellness programme, results in a significant change in:

∘ Number of sick-days off work

∘ BMI

## Methods

A randomised controlled trial was used to evaluate the effect of the employee wellness programme.

### Subject recruitment

Clothing manufacturing companies affiliated to the Clothing Industry Health Care Fund were sent letters explaining the proposed study and invited to participate. The study was also promoted to shop stewards at union meetings. Three out of the eighteen clothing or textile manufacturing companies that were approached agreed to participate in the study. The subjects were drawn from these three clothing industry factories. Participation was open to all employees. Subjects were excluded from the study if they reported uncontrolled diabetes and hypertension, coronary heart disease or any other illness that rendered participation in the exercise component unsafe. Employees were invited to participate in the study through posters and talks which described the study and the exclusion criteria. At each of the three companies approximately 40 employees volunteered for participation in the study. These names were placed in a hat, and thirty employees were randomly selected from each company for inclusion into the study by lottery method [22]. These subjects were then randomly allocated to the control and experimental groups using the same method of randomisation.

### Determining sample size

The primary outcome measure in this study was the EQ-5D quality of life instrument. It consists of items relating to different areas of functioning and a global Visual Analogue Scale (VAS) which requires the respondents to rate their health from 0 (worst possible health) to 100 (best possible health). The sample size was calculated using data on the EQ-5D Visual Analogue Scale (VAS) from a sample of respondents who were similar in cultural background and socio-economic status to the participants of the current study [23]. A sample of 74 was calculated to detect the predicted difference of 11.4 between the means of control and experimental groups with a power level of 90% and the significance level set to 0.05 if the standard deviation was 14.9 and the expected baseline means were 74.7 [23]. The calculated sample size required was 37 in each group.To allow for attrition, a sample size of 90 subjects was selected (30 from each factory).

### Intervention

The Employee Wellness Programme (EWP) consisted of one hour weekly group sessions spread over a period of six weeks. The sessions which were facilitated by a physiotherapist comprised of a 30-minute health promotion talk and a 30-minute exercise class. The talks were structured to promote self-efficacy by teaching participants the skills of goal setting, pacing and self-reflection and were based on the principles of CBT [12]. Each week the workshops addressed a different theme. The themes were pain, back care, chronic diseases of lifestyle, goal setting and pacing, physical activity, nutrition and relaxation. Participants were handed a goal sheet and were guided on how to set individual, specific and realistic goals for themselves. A health promotion pamphlet with information about the session’s topic was given at the end of each session. These were the same pamphlets given to the control group at their once-off session. The exercise class comprised of a brief warm-up, low to moderate intensity aerobics, core stability exercises and a cool down period including stretching and relaxation techniques. The intensity and repetitions of the exercises were gradually increased each week.

The control group received a once-off thirty minute health promotion and motivational talk by a physiotherapist. The talk focussed on back and neck-care and the benefits of regular exercise. Various health promotion pamphlets were given to the control group at this session

### Instrumentation for outcome measurement

#### Independent variables

All study participants had to complete a one page questionnaire recording their demographic information. This included their age, date of birth, gender, preferred language, level of education, current occupation and job description, and years of employment within the clothing industry. Absentee records were obtained for all participants from the human resource officer of each factory.

#### Dependent variables

The outcome measures used in this study to measure the dependent variables included measures of health related quality of life (EQ-5D) and the amount of exercise undertaken (Stanford Exercise Behaviours Scale). The participants’ weight and height were measured and the body mass index (BMI) of the participants was calculated.

#### EQ-5D

The EQ-5D is a self-completion questionnaire that was developed by the EuroQol group. It encompasses two components, the first being a descriptive health state and the second being an evaluation of perceived health [24]. The descriptive health state consists of five domains including mobility, self-care, usual activities, pain/discomfort and anxiety/depression [25]. The second component is the visual analogue scale on which the participants rate their health from the best possible (100) to the worst possible (0) health state imaginable.

#### Stanford exercise behaviours scale

Various definitions of exercise exist. Exercise as defined by Caspersen et al. is “structured and planned physical activity that is aimed at improving fitness levels”[26]. The Stanford Exercise Behaviours Scale measures the amount of exercise performed during the past week. Amount of exercise is captured by minutes or hours engaged in over the last seven days. It consists of five categories, namely stretching/strengthening, walking, swimming, cycling and other aerobic exercise [27].

#### Absenteeism

Rates of absenteesism were based on factory attendance registers and the number of days off work for the six week prior, six weeks during and six weeks after the intervention were recorded. The total number of days of work where recorded for those respective time-frames.

#### BMI

The subjects’ height and weight were measured to determine their BMI. Subjects were weighed on a manual scale. BMI was calculated using the formula below:

(1) $$\text{BMI}=\text{weight}\;\text{in}\;\text{kilograms}÷{\text{height}\;\text{in}\;\text{metre}}^{2}$$

### Procedure

Ethical approval for the study was obtained from the Human Research Ethics Committee of the University of Cape Town. Permission to access the factories was obtained from management. The researcher addressed workers meetings, shop stewarts and supervisors to explain the purpose and nature of the study and to encourage recruitment. Pamphlets were also distributed amongst the workers. The process of selection of participants has been described above. Informed consent was obtained from each participant.

Measurements were recorded for all participants at baseline and six weeks after the initiation of the intervention programme. The person who took the measurements was blinded as to group allocation. Attendance at work was monitored for a further six weeks after cessation of the study.

### Statistical analysis

Statistica version 8 (2008) was used for data analysis. Non-parametric tests were used to analyse the ordinal variables. The Sign test was used to determine whether there was a significant difference between the pre and post-intervention scores within each group (i.e. within group changes). The Mann–Whitney U test was used for between-group comparisons at baseline and at six weeks post-intervention.

## Results

The sample consisted initially of 90 subjects, with the control and experimental groups having 45 subjects each. Six subjects from the experimental group withdrew their consent before baseline questionnaires were completed, leaving the experimental group with 39 subjects. Four of the six subjects withdrew from the study due to personal reasons, while the other two withdrew as their working contract was terminated due to retrenchments at the company. Four subjects from the control group also withdrew consent before baseline measurements were recorded, as their working contracts had also been terminated due to retrenchments at the company. This left the control group with 41 subjects. Data was therefore analysed on 80 subjects.

There was no significant difference between the demographic data of the control and experimental groups (see Table 1). The attendance rate at the classes of the experimental group across the six weeks for the intervention sessions was 94%.

**Table 1:** Demographic characteristics of the experimental and control groups

	**Experimental group:**	**Control group:**	**p-value**
Subjects (N=80)	39 (48.1%)	41 (50.6%)	0.637
Gender: Female	35 (89.74%)	35 (85.37%)	0.554
Age Years (Mean, SD)	37.3 (9.87)	34.8 (11.50)	0.306
BMI mean (SD)	28.90 (6.29)	29.80(9.37)	0.614
Department			0.071
Production	25 (64.1%)	33 (85.4%)	
Administration	4 (35.9%)	6 (14.6%)	

### EQ-5D

At baseline, the most commonly reported problems in both groups were in the domains of pain/discomfort and anxiety/depression and the control group reported more problems overall. There was no significant difference between the two groups at baseline or at six weeks post intervention (see Table 2).

**Table 2:** Self-reported health status (EQ-5D)

**EQ-5D**		**Experimental**		**Control**			
**Domains**		**Group (N=39)**		**Group (N=41)**			
		**Baseline**	**6-weeks post intervention**	**Baseline**	**6-weeks post intervention**	**Baseline**	**6-weeks post intervention**
Mobility	No problems	34	32	33	30	Chi-sq= 0.33	Chi-sq=0.90
	Some problems	5	7	7	11	P= 0.560	P=0.340
Self-care	No problems	37	37	38	38	Chi-sq=0.16	Chi-sq=0.16
	Some problems	2	2	3	3	P=0.686	P=0.686
Usual care	No problems	32	34	37	32	Chi-sq=1.10	Chi-sq=1.15
	Some problems	7	5	4	9	P=0.287	P=0.282
Pain/ Discomfort	No pain/discomfort	24	21	18	19	Chi-sq=2.16	Chi-sq=0.31
	Moderate pain	15	18	22	21	P=0.140	P=0.573
Anxiety/ Depression	Not anxious	31	29	28	27	Chi-sq=0.94	Chi-sq=0.69
	Moderate anxiety	8	10	12	14	P=0.330	P=0.406

The baseline median VAS scores describing the samples perception of their state of health were 80.0 (range 70–90) for the experimental group, and 70.0 (range 60–90) for the control group. At six weeks these scores were 90 (range 70–95) and 80 (55–90) respectively and the difference approached significance (p=0.076). There was a significant within group improvement in the experimental group (*p=0.045*) but not in the control group (p=0.472) (see Table 3).

**Table 3:** Change in EQ-5D VAS scores within each group

**Change in value between baseline and 6-weeks post-intervention score**	**Experimental Group**			**Control Group**		
	**Percent second score higher**	**z**	**p**	**Percent second score higher**	**z**	**p**
VAS	70.0	2.01	***0.045***	58.0	0.72	0.472

### Exercise behaviours scale

No significant differences were seen at either time point between the two groups’ behaviours for all five categories strengthening/stretching, walking, cycling, swimming and other aerobic exercise. Participation in all behaviours except swimming improved from baseline (see Table 4 and Table 5). However, apart from cycling in the control group, these changes were only significant in the experimental group (stretching/strengthening p<.03 in every case).

**Table 4:** Frequency distribution of the exercise behaviours

		**Experimental group (N=39)**		**Control group (N=41)**	
		**Baseline**	**Six weeks post-intervention**	**Baseline**	**Six weeks post-intervention**
Participation in stretching or strengthening exercises	None	25	3	23	19
	Less than 30 minutes/ week	7	19	9	14
	30-60 minutes/ week	5	11	4	5
	1-3 hours/ week	1	6	4	2
	More than 3 hours/ week	1	3	1	1
Participation in walking for exercise	None	13	2	11	7
	Less than 30 minutes/ week	12	9	11	15
	30-60 minutes/ week	9	15	8	9
	1-3 hours/ week	2	9	4	4
	More than 3 hours/ week	3	4	7	6
Participation in swimming for exercise	None	34	31	33	35
	Less than 30 minutes/ week	1	6	4	2
	30-60 minutes/ week	2	2	3	1
	1-3 hours/ week	1	0	1	1
	More than 3 hours/ week	1	0	0	2
Participation in cycling for exercise	None	39	32	37	35
	Less than 30 minutes/ week	0	4	2	1
	30-60 minutes/ week	0	2	1	3
	1-3 hours/ week	0	1	1	0
	More than 3 hours/ week	0	0	0	2
Participation in other aerobic exercise	None	35	23	36	34
	Less than 30 minutes/ week	3	6	1	2
	30-60 minutes/ week	1	6	2	2
	1-3 hours/ week	0	3	2	1
	More than 3 hours/ week	0	1	0	2

**Table 5:** Within group changes for exercise behaviours

	**Experimental group (N=39)**			**Control group (N=41)**		
	**Percent improved**	**z**	**p**	**Percent improved**	**z**	**p**
**Baseline & 6-weeks post intervention participation in strengthening/stretching exercises per week**	87	3.95	**<0.01**	58	0.59	0.556
**Baseline & 6-weeks post intervention participation in walking for exercise per week**	77	2.87	**<0.004**	57	0.57	0.571
**Baseline & 6-weeks post intervention participation in swimming for exercise**	60	0.67	0.546	56	0.00	1.000
**Baseline & 6-weeks post intervention participation in cycling or bicycling for exercise**	100	2.27	**0.023**	71.4	0.76	0.450
**Baseline & 6-weeks post intervention participation in other aerobic exercise**	81	2.25	**0.024**	63	0.35	0.724

### Absenteeism

Absenteeism was assessed for the six week period before the start of the study, six weeks during the study, and the six week period after the completion of the study. There were no significant differences detected between the two groups. However, both groups took less time off work from the beginning to the end of the study.

### Body mass index (BMI)

At baseline the mean BMI was 28.9 (SD= 6.29) for the experimental group and 29.8 (SD=9.37) for the control group. Statistically there was no significant difference in the baseline BMI measurements of the two groups (u=791, z=0.08, p=0.935).

At six weeks post intervention 89% of the experimental group reduced their BMI measurements. (z=3.73, ***p=<0.01)***. In comparison, 79% of the control group reduced their BMI measurements at six weeks post intervention, (z=2.83, ***p=0.05***) (see Table 6). Statistically there was no significant difference in the BMI scores between the two groups at six weeks post intervention.

**Table 6:** Change in BMI scores

	**Experimental group (N=39)**			**Control group (N=41)**		
	**Percent lower**	**z**	**p**	**Percent lower**	**z**	**p**
Change in BMI scores: 6-weeks post intervention-baseline	88.5	3.73	***<0.01***	78.6	2.83	***0.005***

## Discussion

The majority of the participants in this study were female which is representative of the gender profile of both local and global clothing manufacturing employees [28,29]. The age distribution of the sample in this study is similar to the South African economically active population range of 25-55years[30] that allows the results to be generalised to the South African working age population. It may be argued that although the subjects were randomly assigned to the experimental and control groups, the sample comprised of volunteers who were interested in improving their own health and thereby may not be representative of all the employees within the clothing and textile manufacturing industry. Participants in this study were particularly unhealthy with regards to baseline BMI measurements. The mean BMI of the sample was 29.36 at baseline, which is indicative of overweight but closely approaching obesity. This is representative of adults in the Western Cape Province [31].

There was little or no attrition in the number of participants that would have threatened the internal validity of this study. Attendance at the weekly intervention sessions was generally high which further strengthened the study’s internal validity. Similarly, other worksite intervention studies have also reported a high attendance and participation rate but these studies were not randomised controlled trials [32,33]. The high attendance and compliance of participants in this study could partly be attributed to excitement at the companies associated with the EWP as it was the first time an EWP was implemented within the South African Clothing Manufacturing Industry. These findings suggest that clothing industry workers welcome the idea of worksite wellness programmes and are motivated to participate in such programmes.

The outcome of this study demonstrated that the CBT-based EWP implemented over a period of six weeks was no more effective in improving self-reported difficulties on the EQ-5D than the once-off education session. The lack of significant difference between the two groups EQ-5D health domains (mobility, self-care, usual activities, pain, and depression) was not completely unexpected as the short-term EWP was not focused on symptomatic relief, but rather designed to equip employees with the knowledge and skill of improving health and promoting positive health behaviour change. This approach was utilised to afford the participants an opportunity to set realistic short-term goals for themselves and to gradually progress their behaviour changes. Similarly, a study by Baker et al. (1998) [34] showed that participating in a short-term combined walking and education programme did not produce significant changes in the EQ-5D utility scores amongst the participants.

Although we may not have expected large symptomatic improvements in this study, the lack of significant change could also be attributed to the choice of outcome measure. The EQ-5D is a generic instrument that measures health-related quality of life and is not disease specific [23]. Although the EQ-5D has been validated for use among the South African population [35], a few studies have reported it to be insensitive to detect a change when baseline scores are high [36,37]. In this study, the majority of the subjects had high baseline scores for the EQ-5D health state descriptors indicating no problems with most of the functional domains. Therefore, the lack of significance in the change of scores could be attributed to insensitivity of the EQ-5D.

The experimental group’s improvement in EQ-5D VAS scores at six weeks post intervention could be attributed to their participation in weekly exercise and the perceived knowledge gained from the workshops. Individuals with improved levels of self-esteem and good psychological wellbeing are more likely to report better perceived health states [38]. Consistent with this study’s findings, other studies have also reported that a combination of behaviour therapy and supervised exercise programs is effective in improving the perceived health state of patients with musculoskeletal type pain [15,39,40]. In the study by Dahl and Nillson (2001) the cognitive behaviour therapy sessions were administered by a physiotherapist and registered nurse on an individual basis, twice a week for four weeks [15]. The study by Wigers and Finset (2007) showed improved overall HRQoL at six months amongst patients with chronic musculoskeletal pain after participating in a four week CBT education and exercise programme. However, their study did not have a control group and in conjunction with the behavioural intervention the participants also attended individual physiotherapy sessions four times per week for myofascial release.

However, since a ten point improvement was noted in both the control and the experimental group’s EQ-5D VAS scores at six weeks post-intervention, the improvements in perceived HRQoL cannot only be attributed to the EWP. The overall improvement may be due to the Hawthorne or other non-specific effects as a result of being part of the study. The subjects may have perceived their health as improved due to being a participant in a health and wellness study. Long-term follow-up studies are recommended to investigate whether the improvements of perceived HRQoL is maintained after the intervention ends and whether the difference between the two groups becomes more apparent in the long-term.

The EWP encouraged participants to establish weekly goals for themselves, exercise at their own pace and to engage in leisure time physical activities that they enjoyed. This could have contributed to the increase of physical activity behaviours amongst the experimental group. Literature suggests that individual goal setting abilities have a positive effect on short-term behaviour change and exercise maintenance [41,42].

Educating employees on nutrition and cooking methods coupled with supervised exercise programmes appear to have an impact on reducing BMI. The EWP in this study encouraged participants to set weekly nutrition goals, increase their daily fruit and vegetable consumption and advised on healthy cooking methods. This, in conjunction with increased exercise, could explain the reduction in BMI over the short time-frame. Similarly, a study by Aldana et al. (2005) proved that a worksite intervention programme addressing nutritional behaviours and encouraging self-monitoring of nutrition was effective in reducing the BMI of the participants at six weeks post intervention. Although their study did not include participation in supervised aerobic exercise classes [43], their educational workshops were much longer in duration and were more frequently held in comparison to this study.

However, this study also reported a reduction in the control group’s BMI. The control group did receive a once-off health promotion talk and education pamphlets that guided on healthy eating and cooking methods. In addition, all subjects entered the study highly motivated to improve their health. Self-motivation and intention to change has been associated with successful health behaviour change [17]. Overall, these findings suggest that worksite health promotion intervention has an effect on reducing workers BMI.

Similar to the decrease in BMI across the two groups, this study found a statistically significant reduction in sickness related absenteeism of the entire sample group post completion of the study. The overall reduction in sickness related absenteeism could be attributed to improved morale amongst the study participants due to the excitement of the wellness study. The fact that the employers allowed the wellness study to occur during paid working hours may have resulted in a perception that management was interested in their health and wellbeing. According to Nawaz (2006) acknowledging and appreciating employees promotes improved morale at the workplace [44]. The overall reduction in absenteeism in this study indicates that worksite wellness programmes can potentially offer positive economic benefits to the company. However, in order to determine the financial effects a larger population would be required and absenteeism rates would need to be monitored over a much longer period than was done in this study.

### Strengths and limitations

The major strength of the study was the use of a randomized controlled experimental design. The sample size was sufficient to detect a predicted difference. The same person conducted the wellness programme at the three companies and thereby standardization of the intervention programme was maintained.

A major short-coming of the study was that it failed to record the detailed medical history of the participants. It could be argued that the groups were not matched for co-morbidities which could have impacted on results and adherence to the programme. Therefore it is unknown whether the presence of co-morbidities in either group impacted on the results. Apart from the BMI measurements and absenteeism records assessed, the study relied significantly on self-report outcome measures which could have introduced bias. A possible Hawthorne effect may have caused information bias as the participants may have tried to please the researcher by giving favorable and not honest answers. However, considering that this study was primarily concerned with the effects of the intervention on perceived HRQoL and exercise behaviours, self-report measures are the only possible indicators of change. Double-blinding is impossible with this type of intervention as participants can easily identify what is the intervention being tested. A further limitation of the study was that although subjects were randomly selected, the subjects that consented to being part of the study were all motivated and interested in improving their health. Therefore it remains unknown whether the EWP would have the same effect on subjects who are not motivated to change their behaviours.

## Conclusion

To our knowledge this wellness study was the first of its kind performed within the South African clothing manufacturing industry. The findings of the study report that the EWP appeared to be beneficial in improving health-related behaviours and perception of health-related quality of life of the employees. However, it cannot be concluded that the EWP was more effective than the once off health promotion talk as no significant changes were noted between the two groups at 6-weeks post intervention. It is recommended that similar programmes be introduced and then monitored in other factories in the Western Cape.

## Abbreviations

(LBP): Lower back pain; (RSI): Repetitive Strain Injury; (CBT): Cognitive behaviour therapy; (VAS): Visual Analogue Scale; (EWP): Employee Wellness Programme; (BMI): Body Mass Index.

## Competing interests

We declare competing interests. The University of Cape Town places physiotherapy students at the factories in question to gain clinical experience. The corresponding author supervises these students. In recognition of the services provided by the students, payment is made by the factories to the University of Cape Town.

## Author information

All three authors are physiotherapists and employed at an academic institution. NE is a specialist in musculoskeletal physiotherapy and ergonomics, JJ is particularly interested in determinants of health related quality of life and SM works in the field of community health and disability.

## Authors’ contributions

All three authors contributed to the conceptualisation, analysis and write up of the paper. NE was responsible for data collection. All authors read and approved the final manuscript.

## Pre-publication history

The pre-publication history for this paper can be accessed here:

http://www.biomedcentral.com/1471-2458/13/25/prepub
